# Construction of a biodynamic model for Cry protein production studies

**DOI:** 10.1186/s13568-014-0079-y

**Published:** 2014-11-14

**Authors:** Ana Karin Navarro-Mtz, Fermín Pérez-Guevara

**Affiliations:** Instituto de Biotecnología, Universidad del Papaloapan, Circuito Central 200, Parque Industrial, Oaxaca, 68301 Tuxtepec, México; Departamento de Biotecnología, Centro de Investigación y de Estudios Avanzados, Mexico City, 07000 México

**Keywords:** Biodynamic model, Culture dynamics, Bacillus thuringiensis, Cry production, Poly-β-hydroxybutyrate

## Abstract

Mathematical models have been used from growth kinetic simulation to gen regulatory networks prediction for *B. thuringiensis* culture. However, this culture is a time dependent dynamic process where cells physiology suffers several changes depending on the changes in the cell environment. Therefore, through its culture, *B. thuringiensis* presents three phases related with the predominance of three major *metabolic pathways*: vegetative growth (Embded-Meyerhof-Parnas pathway*)*, transition (γ-aminobutiric cycle) and sporulation (tricarboxylic acid cycle). There is not available a mathematical model that relates the different stages of cultivation with the metabolic pathway active on each one of them. Therefore, in the present study, and based on published data, a biodynamic model was generated to describe the dynamic of the three different phases based on their major metabolic pathways. The biodynamic model is used to study the interrelation between the different culture phases and their relationship with the Cry protein production. The model consists of three interconnected modules where each module represents one culture phase and its principal metabolic pathway. For model validation four new fermentations were done showing that the model constructed describes reasonably well the dynamic of the three phases. The main results of this model imply that poly-β-hydroxybutyrate is crucial for endospore and Cry protein production. According to the yields of dipicolinic acid and Cry from poly-β-hydroxybutyrate, calculated with the model, the endospore and Cry protein production are not just simultaneous and parallel processes they are also competitive processes.

**Electronic supplementary material:**

The online version of this article (doi:10.1186/s13568-014-0079-y) contains supplementary material, which is available to authorized users.

## Introduction

*Bacillus thuringiensis* (*B. thuringiensis*) is a Gram-positive endospore-forming bacterium isolated from soil that synthesizes a crystalline δ-endotoxin protein, named Cry protein (Ito et al. [2004]). The main application of the Cry protein is the biological control of certain insects of several orders (Ito et al. [2004]). It has been also reported that some non-toxic Cry protein for insects are highly cytotoxic to a wide range of mammalian cells, particularly human cancer cells (Ito et al. [2004]). Cry protein production by *B. thuringie* nsis has been largely studied. Many different culture media formulations, carbon-nitrogen ratios, operating conditions and production systems have been used in order to improve the cost-productivity relation (Farrera et al. [1998]; Navarro et al. [2006]; Ozcan et al. [2010]; Zhuang et al. [2011]). Also, several mathematical models describing the growth and endospore formation kinetics of *B. thuringiensis* are available (Kraemer-Schafhalter and Moser [1996]; Liu and Tzeng [2000]; Popovic et al. [2001]; Rivera et al. [1999]; Starzak and Bajpai [1991]).

**Figure 1:**
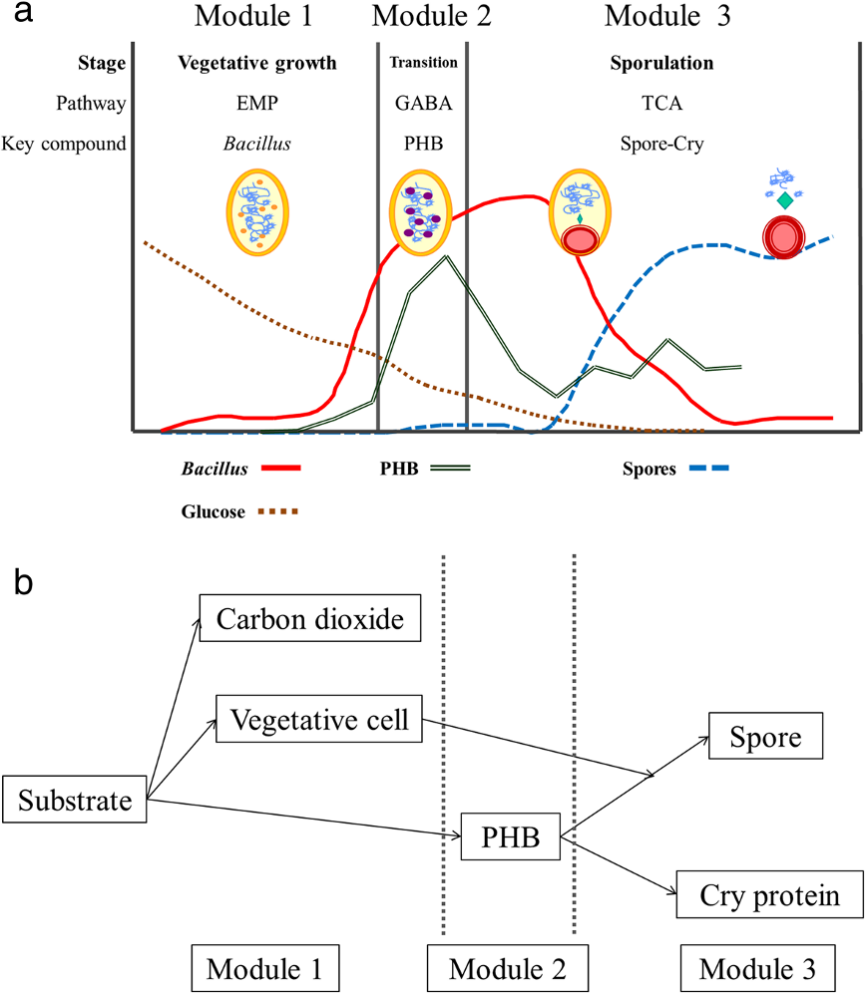
**Schematic representation of (a)** ***B. thuringiensis*** **culture with their key compounds and (b) model modules.**

*B. thuringiensis* culture is a time dependent dynamic process where the physiology of cells suffers several changes. During the culture the number of cells increase causing changes in the environment and subsequently the cells respond to those changes modifying their activity. This dynamics continues until the death of the cells when active entomopathogenic products (endospores and Cry protein) are obtained. The response of the cell to the environmental changes is a complicated process that involves cell metabolism, cell cycle regulation, signaling and gene regulatory networks and gene expression. As result of the response of the cell to the environmental changes, the culture typically shows three different phases related with the predominance of one of three major *metabolic pathways* (Figure 1a): vegetative growth-*Embded-Meyerhof-Parnas pathway* (EMP), transition-*γ-aminobutiric cycle* (GABA) and sporulation-*tricarboxylic acid cycle* (TCA) (Anderson [1990]; Rowe [1990]).

Mathematical models are powerful tools to describe processes or natural systems that are reproducible and predictable, which also simplify reality. Models are designed to focus on certain aspects of the system of study while other aspects are abstracted away. The mathematical modeling serves as aids to biological investigation in a number of ways. Different models have been used to describe the different phases present in *B. thuringiensis* culture, specifically: the vegetative growth phase (Kraemer-Schafhalter and Moser [1996]; Popovic et al. [2001]; Rivera et al. [1999]) and the sporulation phase (Liu and Tzeng [2000]). These models consider just two phases of the culture and represent only one or another. Starzak and Bajpai et al. ([1991]) model considers the growth and the sporulation phases but it assumes that during sporulation there are no energy requirements so no link between both phases is considered. Thus, there is no mathematical model which links the different culture phases with its metabolic pathway.

In the present study, a biodynamic model was constructed to describe the complete evolution of the *B. thuringiensis* culture. Also, it was used to study the interrelation between the different culture phases and its relationship with the Cry protein production. The considerations for the construction of the model were: a) each phase of *B. thuringiensis* culture can be represented by its principal metabolic pathways (EMP for vegetative growth, GABA for transition and TCA for sporulation); and b) each pathway can be follow with its key compound (cell for EMP, PHB for GABA and DPA-Cry protein for TCA).

## Materials and methods

### Model development

The previously reported kinetics of the key compounds were analyzed to identify the main variables affecting the three phases of *B. thuringiensis* culture. It is not straightforward to compare the cells/biomass and PHB kinetics data reported in the literature because different conditions and analytic techniques were used. Thus, in order to be able to compare these data a normalization was done (Additional file 1). The normalization is the mathematical process used to reduce the data to absolute values. The normalized cells/biomass and PHB kinetics show the sigmoidal curve typically well represented by the Gompertz model. Also, the two sets of endospores’ kinetics data published (Farrera et al. [1998]; Navarro et al. [2008]) show a sigmoidal curve. Therefore, the Gompertz model was used to simulate and estimate the kinetic parameters of the key compound data.

### Model formulation

The assumptions for the biodynamic model construction were: i) PHB affects positively the Cry production; ii) Yield of DPA, Cry protein and PHB from biomass are independent from initial substrate concentration; iii) Embded-Meyerhof-Parnas, γ-aminobutyric acid cycle and tricarboxylic acid cycle are the main pathways for vegetative growth, transition and sporulation phases, respectively; iv) Every metabolic change has a key compound associated and can be represented by: cells, poly-β-hydroxybutyrate (PHB) and DPA-Cry protein.

As show in Figure 1b the biodynamic model has three principal modules interconnected, one for each phase. Module 1 represents the vegetative growth, module 2 represents the transition and module 3 represents the sporulation phase. In general terms, during vegetative growth the substrate is used to generate new cells and carbon dioxide. The CO_2_ is released due to the respiration process. During transition the substrate is accumulated as PHB. While during sporulation: there is no substrate consumption; PHB is consumed; the vegetative cell is transformed into endospore; and in the same period of time the Cry protein is produced from protein turnover (Anderson et al. [2002]). The PHB is the energy source for endospore and Cry protein formation processes. Thus, module 1 and module 2 are related with module 3.

#### Module 1

Phase: Vegetative growth; metabolic pathway: Embded-Meyerhof-Parnas pathway; key compound: cells. The vegetative cell evolution requires a two-term equation: growth and dead (Equation 1). The first term is simulated using the Gompertz model while the second term takes into account that endospores (DPA) are formed from biomass. The meanings of the symbols were defined in Table 1.

1 $$X=X_{\max}e^{\left(-e\left(-u_{\max}\left(t-t_{c}\right)\right)\right)}-\left[X_{\max}-\frac{\mathrm{DPA}}{Y_{\mathrm{DPA}/X}}\right]$$

**Table 1:** **Nomenclature**

Parameter	Description	Unit
Cry	Cry protein concentration	g/L
Cry_max_	Maximum Cry protein concentration obtained	g/L
DPA	Dipicolinic acid concentration	g/L
DPA_max_	Maximum dipicolinic acid concentration obtained	g/L
PHB	Poly-β-hydroxybutyrate concentration	g/L
PHB_max_	Maximum Poly-β-hydroxybutyrate concentration obtained	g/L
t	Time since inoculation	h
tc	Critical time	h
X	Biomass concentration	g/L
X_max_	Maximum biomass concentration obtained	g/L
Y_Cry/PHB_	Yield of Cry from PHB	g Cry/g PHB
Y_DPA/PHB_	Yield of DPA from PHB	g DPA/g PHB
Y_DPA/X_	Yield of DPA from biomass	g DPA/g X
Y_X/S_	Yield of biomass from glucose	g biomass/g glucose
μ_max_	Maximum specific growth rate	1/h
μ_maxc_	Maximum specific Cry production rate	1/h
μ_maxd_	Maximum specific DPA production rate	1/h
μ_maxp_	Maximum specific PHB production rate	1/h
**Subindex**		
c	Cry protein	
d	Dipicolinic acid	
i	Key compound (i = x, p, d or c)	
p	Poly-β-hydroxybutyrate	
x	Biomass	

#### Module 2

Phase: Transition; metabolic pathway: γ-aminobutyric acid cycle; key compound: Poly-β-hydroxybutyrate. The PHB also requires a two-term equation: production and consumption (Equation 2). In the first term, PHB production was modeled using the Gompertz model. It has been reported that the PHB serves as an endogenous reserve of carbon and energy during the sporulation and it is necessary for the δ-endotoxin synthesis (Benoit et al. [1990]; Kraemer-Schafhalter and Moser [1996]; Navarro et al. [2006]; Rowe and Margaritis [1987]). Therefore, the second term takes into account that the production of DPA and Cry is based on the consumption of PHB.

2 $$PHB=PHB_{\max}e^{\left(-,e,\left(\mu {\mathrm{maxp}}^{\left(t-tc_{p}\right)}\right)\right)}-\left[PHB_{\max}-\frac{\mathrm{DPA}}{Y_{\mathrm{DPA}/\mathrm{PHB}}}-\frac{\mathrm{Cry}}{{Y_{\mathrm{Cry}/}}_{{}_{PHB}}}\right]$$

#### Module 3

Phase: Sporulation; metabolic pathway: tricarboxylic acid cycle; key compound: Cry protein and DPA. DPA and Cry protein production are assumed as simultaneous and parallel processes both represented by the Gompertz model, Cry protein (Equation 3) and DPA (endospore) (Equation 4):

3 $$\begin{array}{l} \mathrm{Cry}=Cry_{\max}e\left(-e^{(-\mu_{\mathrm{maxc}}\left(t-tc_{c}\right)}\right) \end{array}$$

4 $$DPA=DPA_{\max}e\left(-e^{(-\mu_{\mathrm{maxd}}\left(t-tc_{d}\right)}\right)$$

The biodynamic model constructed (Equations 1-4) was solved with ModelMaker 3.0.3. The model parameters were estimated by nonlinear minimum sum of squares analysis (i.e. the differences between the predicted and measured values) using the Marquardt algorithm.

The biodynamic model was constructed using the kinetic data sets available in the literature and the model validation was done by the comparison of the predicted values and those obtained in four new experiments.

### New data sets for model validation

The biodynamic model validation was made using the data of the key compounds kinetics obtained in four new fermentations (F1, F2, F3, F4). These fermentations were done using the same culture media, carbon:nitrogen relation, operating condition and bioreactor. In order to establish the relationship between the carbon source and the biomass and Cry protein production, different concentration of the substrates in the culture media were used in each run.

#### Organism

*Bacillus thuringiensis* var. *kurstaki* HD-73 (ATCC-35866), which produces a 133.3 kDa Cry1A(c) insecticidal crystal protein, was obtained from the USDA Insect Pathology Research Unit, Brownsville, Texas. The microorganism conservation was made in endospore filter-paper disk and the inoculum for fermentation was prepared in two stages, according to Navarro et al. ([2006]). The culture of the second stage was used as inoculum for the bioreactor experiments.

#### Fermentation procedures

All the fermentations were done in a 7 L glass reactor. The reactor was fully automated and equipped with two Rushton turbines. The operating and the sterilization conditions were done according to Navarro et al. ([2006]). The end-of-fermentation criterion was set to 85-90% of endospores released determined microscopically. Samples were collected every 2 h during the fermentation.

#### Media

The media was adjusted for all the fermentations to a carbon:nitrogen ratio of 7:1; to achieve this ratio several glucose-soybean meal concentrations were used (Table 2). Additionally, all media contains: 5.8 g/L yeast extract, 9.2 g/L corn steep liquor, 3 g/L KCl, 0.2 mg/L MgSO_4_ • 7H_2_O, 40 mg/L MnSO_4_, 30 mg/L CoCl_2_, 7.5 mg/L CuSO_4_ • 5H_2_O, 7.5 g/L ZnSO_4_ • 7H_2_O, 1.35 mg/L FeSO_4_ and 7 ml/L H_3_PO_4_ 85% (w/v).

**Table 2:** **Parameters estimated with structural model**

Parameter	F1	F2	F3	F4
Glucose-Soybean meal (g/L)	25.1-4.4	34.74-14.73	44.42-25.05	54.1-35.37
X_max_ (g/L)	5.58	8.58	10.09	13.07
PHB_max_ (g/L)	0.61	0.71	1.16	1.41
DPA_max_ (g/L)	0.17	0.18	0.16	0.16
Cry_max_ (g/L)	0.19	0.31	0.53	0.78
μ_max_ (1/h)	0.78	1.06	0.90	0.86
μ_maxp_ (1/h)	1.07	0.89	0.95	1.02
μ_maxd_ (1/h)	0.66	0.72	0.59	0.65
μ_maxc_ (1/h)	0.20	0.42	0.40	0.33
t_c_	4.54	4.12	4.95	6.58
t_cp_	8.46	7.90	8.39	8.88
t_cd_	14.12	12.94	15.77	14.32
t_cc_	14.88	12.77	14.96	16.46
Y_DPA/X_ (g DPA/g biomass)	0.025	0.030	0.018	0.0093
Y_DPA/PHB_ (g DPA/g PHB)	1.02	0.43	0.21	0.15
Y_Cry/PHB_ (g Cry/g PHB)	0.19	2.53	4.92	5.83

### Analytical methods

#### Bacillus and endospore count

The counting of cells and endospores was done in a Neubauer chamber with a Nikon Eclipse 55i microscope (Nikon, Tokyo, Japan) under dark field illumination, with a 40 X objective and 10 X ocular lenses, as previously described (Navarro et al. [2008]). In order to express the number of cells as biomass concentration it was consider that the weight of one *B. thuringiensis* cell is 2.3 pg (Rodríguez and de la Torre [1996]).

#### Poly-β-hydroxybutyrate

The PHB concentration was determined in a FID gas chromatograph (SRI Instrument, SRI 8610C, USA) equipped with an AT-1000 column (Navarro et al. [2006]). Each value reported is the average of four replicates. The PHB content, reported as percentage of dry cell weight was calculated with the maximum PHB and biomass concentration obtained in each culture.

#### Cry protein

The concentration of Cry protein was determined by sodium dodecyl sulfate polyacrylamide gel electrophoresis (SDS-PAGE). Bovine serum albumin (BSA) was used as protein standard. The samples preparation was done according to Navarro et al. ([2006]). The solubilization process was done using the method described by Farrera et al. ([1998]). Each value reported is the average of four replicates. The Cry percentage of dry cell weight was calculated with the maximum Cry and biomass concentration obtained in each culture.

#### Dipicolinic acid

Pyridine-2,6-dicarboxylic acid (dipicolinic acid-DPA) is a unique constituent of all endospores of *Bacillus* and *Clostridium* genus. In order to obtain more accurate result, endospore kinetics was follow by DPA photoluminescence technique (Navarro et al. [2008]). The photoluminescence of all samples were measured by duplicate in a spectrofluorometer (SLM 48000C Instrument, Urban IL).

## Results

The normalized data from the literature show that the cells/biomass and PHB kinetics (Additional file 1: Figures S1 and S2) always have the same behavior and that they are independent to: culture systems, operating conditions, culture media, bioreactors used or the techniques used to measure. The Gompertz model was statistically sufficient to describe the growth and PHB data for all previously reported kinetics (Additional file 1).

### Experimental key compounds kinetics analysis for model validation

#### Vegetative growth phase

**Figure 2:**
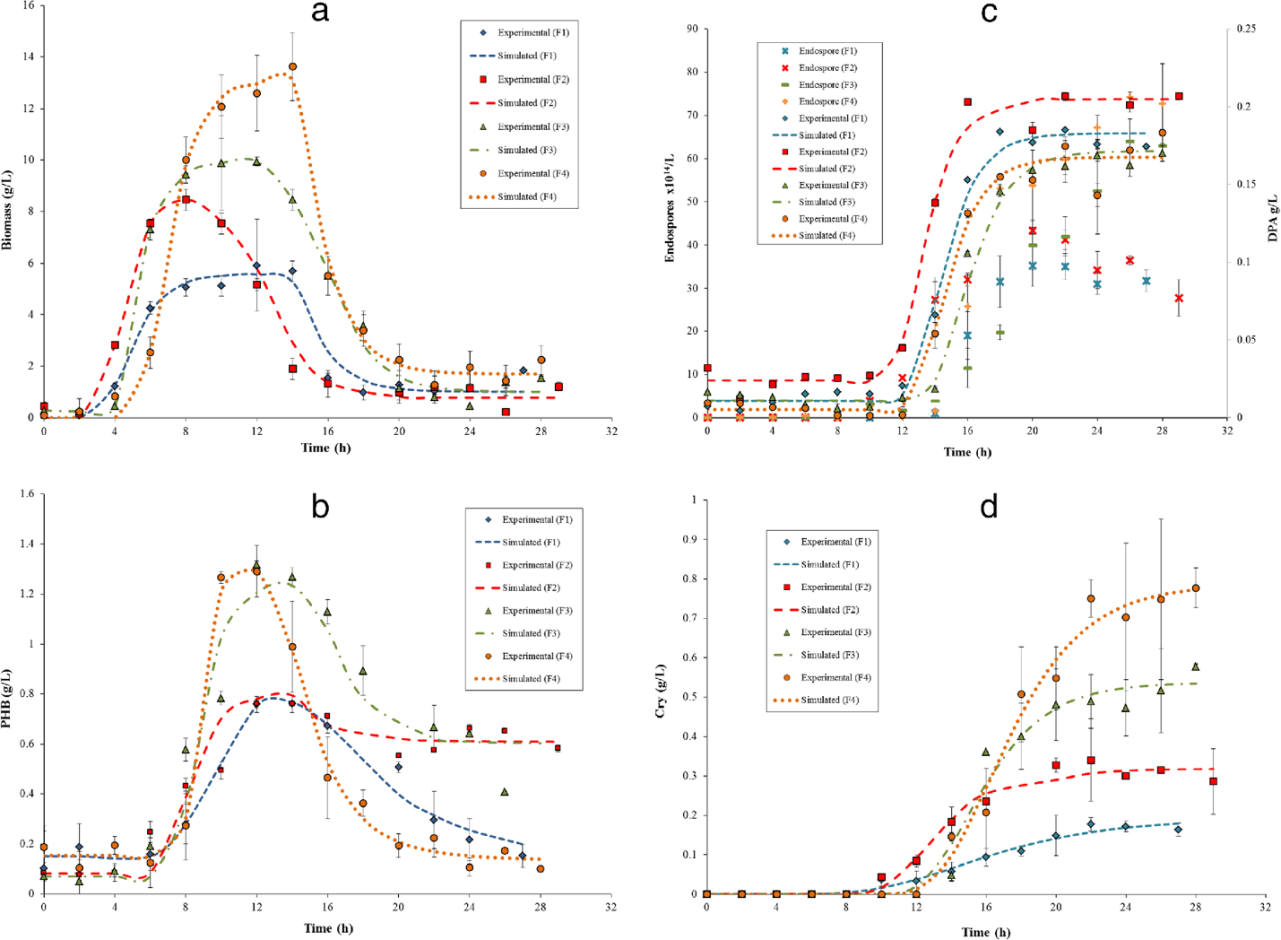
**Experimental and simulated kinetics of key compounds of** ***B. thuringiensis*** **var.** ***kurstaki*** **HD-73.** Key compound kinetics: **(a)** biomass; **(b)** Poly-β-hidroxybutyrate; **(c)** Endospores and dipicolinic acid (DPA); and **(d)** Cry protein. Experimental kinetics of the key compounds (dots) obtained at different glucose and soybean meal concentration. Simulated kinetics of key compounds calculated with the biodynamic model (dotted lines).

The cell kinetics for *B. thuringiensis* var. *kurstaki* HD-73 cultivations are shown in Figure 2a. The cell kinetics show the different phases of the culture (Figure 2a): lag phase (first 3 ± 1 h of culture), vegetative growth (from 3 ± 1 h to 7 ± 1 h of culture), transition (from 7 ± 1 h to 9 ± 1 h of culture) and sporulation (after 9 ± 1 h of culture). The productivity from the experimental data and from the literature in batch culture is shown in Table 3.

**Table 3:** **Parameters culture of** ***Bacillus thuringiensis*** **reported in the literature**

Biomass	PHB	Cry	μ_max_^a^	Y_X/S_^b^	Y_Cry/X_^c^	Comment	Reference
8.41 to 32.19 g/L		0.16-1.24 g/L	0.19 - 1.09	0.43 - 0.67	0.02 to 0.03	Same culture conditions and techniques to this work.	Previous experiments^g^
6.78 to 25.70 g/L^f^	0.52-1.1 mg/L	0.1-1.2 g/L	0.37 - 0.63^f^	0.39 - 0.68^f^	0.01 to 0.04	Same culture conditions and techniques to this work.	Navarro et al. ([2006])
2.57 to 13.64 g/L	8.99 to 13.17%^e^	0.17-0.77 g/L	0.78 - 1.06	0.16 - 0.25	0.06 to 0.05	Culture medium with different initial nutrient concentration. Biomass detection by direct counts technique. PHB detection by CG Technique. Cry detection by SDS-PAGE technique.	Present work
1.65x10^9^ CFU/cm^3^ 3.79 g/L^d^		2.67 g/L			0.7	Starch processing wastewater medium. Biomass detection by CFU technique. Cry detection by SDS-PAGE technique.	Chang et al. ([2008])
16 g/L		12.8 - 15.7 g/L	0.69-1.2		0.8 to 0.98	Fed Batch with balanced flux of substrate. Biomass detection by UV-Vis technique. Cry detection by ELISA technique.	Anderson and Jayaraman ([2005])
3.8x10^8^ CFU/cm^3^^i^ 0.87 g/L^d^		1.043 g/L^i^			1.19	Culture with SodAcet as pH control agent. Biomass detection by CFU technique. Cry detection by alkaline solubilization and Bradford method.	Dang Vu et al. ([2009])
0.34 to 1.02 g/L	7.54 to 29.41%^e^					Nutrient broth as culture medium. PHB detection by UV-Vis technique.	Aslim et al. ([2002])
2.78-3.3 g/L		0.28 - 0.415 g/L				Different oxygen supply. Biomass detection by dry weight technique. Cry detection by alkaline solubilization and Lowry method.	Avignone-Rossa et al. (1992)
5.6-20 g/L^h^			0.58-0.8	0.3-0.7		Culture medium with different initial nutrient concentration. Culture under similar conditions to this work.	Amicarelli et al. (2010)
1.64-4.78 g/L						Culture media with different peptone + yeast extract concentration. Biomass detection by dry weight technique.	Prabakaran and Hoti (2008)
			0.81	2.25^g^		Culture under conditions of carbon limitation.	Popovic et al. ([2001])
			0.58-0.8	0.37-0.7		Culture in intermittent fed-batch with total cell retention. Culture medium with different glucose and yeast extract concentration.	Atehortúa et al. (2007)
8.5 - 15.9 g/L			0.79-1.1	0.41-0.8		Similar conditions of culture to this work. Biomass detection by UV-Vis.	Berbert-Molina et al. ([2008])
		0.36-2.66 g/L				Similar conditions of culture to this work.	Farrera et al. ([1998])
			0.95			Similar conditions of culture to this work.	Holmberg and Sievänen ([1980])
			0.53			Similar conditions of culture to this work.	Rivera et al. ([1999])

^a^Maximum specific growth rate (1/h).

^b^Yield of biomass from glucose (g biomass/g glucose).

^c^Yield of Cry from biomass (g Cry/g biomass). This value was calculated with the biomass and Cry protein concentration reported.

^d^This value was calculated considering that the weight of one *B. thuringiensis* cell is 2.3 pg (Rodriguez and de la Torre, 1996).

^e^PHB percentage of dry cell weight.

^f^Unpublished data.

^g^Yield of active biomass from limiting substrate.

^h^This value was calculated with the glucose concentration in the media and the yield of biomass from glucose reported by the author.

#### Transition phase

The PHB kinetics for *B. thuringiensis* var. *kurstaki* HD-73 cultivations are shown in Figure 2b. During the first 6 ± 1 h of culture no PHB was detected, then its accumulation begins and reaches a maximum at 11 ± 1 h. After this time, PHB concentration gradually diminishes until the end of the culture, this means that the PHB consumption outstrips its production (Figure 2b). The maximum production of PHB in the batch culture of *B. thuringiensis* is far away from the highest production values but agrees with those reported in similar experiments (Table 3).

#### Sporulation phase

The endospore and DPA kinetics for *B. thuringiensis* var. *kurstaki* HD-73 cultivations are shown in Figure 2c. From the first 10 to 14 hours of culture no endospore or DPA formation were detected. Then, the endospore and DPA are accumulated until the end of the culture (Figure 2c).

The kinetics of Cry protein during *B. thuringiensis* var. *kurstaki* HD-73 cultivations are shown in Figure 2d. From the first 10 to 12 hours of culture there is no Cry formation. Then, the Cry protein is accumulated until the end of the culture (Figure 2d). At the same time, cells are dying, so active biomass diminish, there is no carbon source consumption and the PHB is consumed (Figure 2).

#### Biodynamic model validation

**Figure 3:**
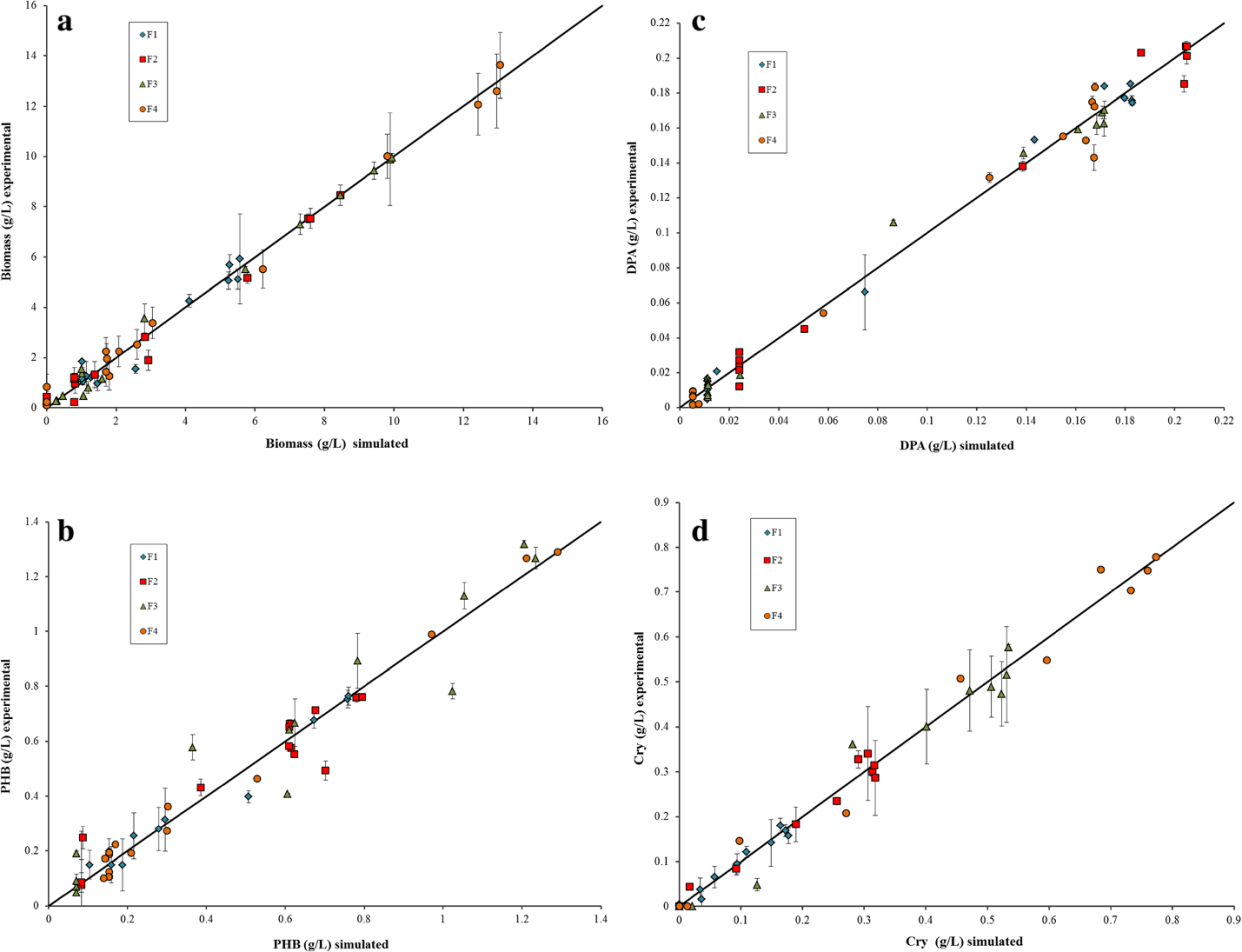
**Comparison of simulated and experimental key compound concentrations of** ***B. thuringiensis*** **var.** ***kurstaki*** **HD-73.** Batch fermentations at different glucose and soybean meal concentration: F1 at 25.1 & 4.4 g/L; F2 at 34.74 & 14.73 g/L; F3 at 44.42 & 25.05 g/L; and F4 at 54.1 & 35.37 g/L, respectively. Comparison between simulated and experimental data of **(a)** biomass; **(b)** Poly-B-hydroxybutyrate; **(c)** dipicolinic acid (DPA); and **(d)** Cry protein.

The model results fit well the experimental data (Figure 2). In general, biodynamic model predicts the kinetics of all selected key compounds (X, PHB, Cry, DPA). The comparison between the experimental and the simulated data is shown in Figure 3a,b,c,d the deviation between them is within the experimental deviations values and they can be fitted with a straight line. The coefficient of determination for all kinetics was always greater than 90%. The parameters obtained with the biodynamic model constructed are listed in Table 2. For biodynamic model validation it is very important that the difference between the values of the parameters obtained theoretically and experimentally were near to zero.

## Discussion

### Key compounds productivity

In general, the experimental data of the biomass and PHB production agree with those previously reported. Avignone-Rossa and Mignone ([1995]) reported the highest cells productivity for *B. thuringiensis* (1.6 × 10^10^ CFU/ml or 36.8 g/L, Table 3) up to date. However, this concentration was obtained in a Fed-Batch system. Pal et al. ([2009]) and Rohini et al. ([2006]) reported two of the highest percentages of PHB per gram dry weight of *B. thuringiensis* cells, 60.3% and 64.1% respectively (Table 3). But, it is worth to mention that these experiments were conducted mainly to improve PHB production.

The sequence of endospore development in *B. thuringiensis* var. *kurstaki* is a seven stages process (Bechtel and Bulla [1976]).

Stage I starts at the beginning of the sporulation process (t_0_); stage II, the first hour; stage III, 1 h after t_0_; stage IV, 2 h after t_0_; stage V, 4 h after t_0_; stage VI, 5 h after t_0_; and stage VII, more than 5 h after t_0_. Figure 2c shows that, in general, the DPA is detected two hours earlier than endospore. This would be expected because during stage V, DPA synthetase is encoded by spoVF (Paidhungat et al. [2000]), and after stage VII, mature endospores are released (Bechtel and Bulla [1976]). During sporulation process, cells are dying, viable cells decrease, the PHB is consumed, but there is no carbon source consumption (Figure 2). Therefore, the PHB becomes the carbon and energy source for Cry protein production as it have been suggested by Benoit et al. ([1990]), Kraemer-Schafhalter and Moser ([1996]), Navarro et al. ([2006]) and Rowe and Margaritis ([1987]). Figures 2c and d show that, in general, the Cry protein is detected before than endospore. This would be expected because the Cry protein is first observed during stage III and the crystal protein is almost full-sized during stage IV of sporulation (Bechtel and Bulla [1976]).

According to the experimental results, the Cry protein represents approximately 6% of the dry cell weight. This result is below some previously reported values (Table 3). However, there is not enough reports using SDS-PAGE for Cry protein quantification and therefore it is difficult to establish a comparison. The Cry protein concentrations reported in literature are scattered and confused. For example, while Rowe and Margaritis ([1987]) reported that after cell lysis the endospore and Cry protein mass were 15% of the biomass dry weight, Agaisse and Lereclus ([1995]) reported that Cry protein can account for up to 25% of the dry weight of the sporulated cells. Even some values from the literature of Cry protein concentration are much higher, for example: Anderson and Jayaraman ([2005]) reported 98% of the biomass dry weight (calculated from the reported values of 15.7 g/L of Cry protein from 16 g/L of biomass, Table 3). Considering these data, the yields coefficient of Cry from biomass are 0.98 g Cry/g biomass (Table 3), which means that almost every cell is transformed into Cry protein. In the same way, the yield coefficient of Cry from biomass obtained by Chang et al. ([2008]) and Dang Vu et al. ([2009]) seems excessively high (Table 3).

### Biodynamic model parameters

The theoretical values of the parameters like the maximum concentrations of the key compounds (X_max_, PHB_max_ and Cry_max_) agree with the experimental and the reported ones (Tables 2 and 3, Additional file 1: Figures S3 and S5). Different maximum specific growth rate (μ_max_) and growth yield coefficients (Y_X/S_) have been reported for different culture conditions (Table 3). For example, Popovic et al. ([2001]) reported a growth yield coefficient of 2.25 g active biomass/g limiting substrate (assuming glucose as the limiting substrate). According to the stoichiometry for anaerobic growth, the maximum Y_X/S_ expected would be 0.863 g biomass/g glucose base on the general chemical composition of biomass (CH_1.8_O_0.5_N_0.2_), 1 C-mol of glucose (CH_2_O) and no respiration process (no formation of CO_2_). Then, the growth yield coefficient for aerobic growth must be always below 0.863 g of biomass/g glucose and values greater than 0.863 imply that glucose is not the limiting substrate. In the present study, the μ_max_ and the Y_X/S_ values obtained agree with previously reported (Tables 2 and 3). The X_max_, PHB_max_, DPA_max_, Cry_max_, tc and tc_p_ relationship with the initial glucose concentration were analyzed from the four new fermentations. The X_max_, PHB_max_ and Cry_max_ have a linear relationship with the glucose concentration in the media, but no relationship with DPA_max_ was found (Table 2). The lag period of each key compound (indirectly represented by tc) shows a linear relationship with the glucose concentration in the media (Table 2). The same behavior was observed with literature data. The tc_x_ y tc_p_ obtained with the model for fermentation in literature with high glucose concentration in formulation is greater than those from fermentation with low concentration (Additional file 1: Figure S4 and S6). There is no relationship between the specific rates of the key compounds with their maximum concentrations (Table 2).

### Dynamics of *B. thuringiensis* culture

The biodynamic model represents the evolution of the *B. thuringiensis* fermentation and it was used to study the dynamic of the process. The three phases of the fermentation are related between them and the Cry protein production is the result of the interaction between those phases. Therefore, the endospore formation is related with the vegetative growth and the endospore-Cry formation is related with the transition phase. This means that the bacilli disappearance is related with DPA (endospore) formation through Y_DPA/X_. In the same way, the PHB consumption is related with DPA-Cry production through Y_DPA/PHB_ and Y_Cry/PHB_. These yields were calculated with the model (Table 2) and they show a different relationship with the glucose concentration in the media. The Y_Cry/PHB_ increases and the Y_DPA/PHB_ decreases when the initial nutrient concentration increases. These results suggest that endospore formation consumes preferably the PHB and excess (if any) is used in the Cry protein formation. There is not data available in the literature to compare these results and further experiments have to be done. Also, the biodynamic model simulation results indicate that the Cry protein production is a function of development of the three fermentation phases, and that endospore and Cry protein production are competitive processes. Therefore, the optimization of each phase cannot be done independently. The reports in the literature usually do not take into account the transition phase neither the PHB production as important variables for Cry production. However, the biodynamic model implies that PHB is crucial for endospore and Cry protein production.

In summary, the new biodynamic model reported here represents well the dynamic of the *B. thuringiensis* fermentation and it can be used as a tool to understand the complex processes related to the physiology of the bacilli. Besides, it can be applied for fermentation with different production systems, operating conditions and culture media. Also, it can be used to study other dynamic processes that occur during fermentation as the cell cycle regulation, the signaling and gen regulatory networks and the changes in gene expression, between others. Although, the biodynamic model is the first model that represents the entire fermentation of *B. thuringiensis* and it is also the first one that links each phase of the culture with its predominantly active metabolic pathway. This model is a powerful tool to study the dynamics of the *B. thuringiensis* culture and the interrelation between their phases. Also, the model can be used to know the physiological phase during the fermentation. From the simulation results it can be concluded that the PHB is crucial for endospore and Cry protein production; and that those processes are simultaneous, parallel and competitive. In terms of the DPA and Cry yield from PHB, the endospore formation is preferred over the Cry protein synthesis.

## Additional file

## Electronic supplementary material

Additional file 1: **Normalization of cells/biomass and PHB kinetics from** ***B. thuringiensis*** **culture. Figure S1.** Normalization of the biomass kinetics from reported data. The data correspond to different culture systems, different operating conditions, different culture media, different bioreactors, etc., and biomass concentrations were measured using different techniques. The line represents an average biomass kinetic for *B. thuringiensis* var. *kurstaki* HD-73. **Figure S2.** Normalization of the Poly-β-hidroxybutyrate kinetics from reported data. The data correspond to different culture systems, different operating conditions, different culture media, different bioreactors, etc. and Poly-β-hidroxybutyrate concentrations were determined using different techniques. The line represents an average PHB kinetic for *B. thuringiensis* var. *kurstaki* HD-73. **Figure S3.** Effect of the glucose concentration on the Xmax for *B. thuringiensis* culture (bibliographic data). **Figure S4.** Effect of the glucose concentration on the tcx for *B. thuringiensis* culture (bibliographic data). **Figure S5.** Effect of the glucose concentration on the PHBmax and for *B. thuringiensis* culture (bibliographic data). **Figure S6.** Effect of the glucose concentration on the tcp for *B. thuringiensis* culture (bibliographic data). (PDF 9 MB)

Below are the links to the authors’ original submitted files for images.

Authors’ original file for figure 1

Authors’ original file for figure 2

Authors’ original file for figure 3

Authors’ original file for figure 4

Authors’ original file for figure 5

Authors’ original file for figure 6

Authors’ original file for figure 7

Authors’ original file for figure 8

Authors’ original file for figure 9
