# Supplementary material for: Construction of a biodynamic model for Cry protein production studies
Source: AMB Express. 2014 Nov 14;4:79. doi: 10.1186/s13568-014-0079-y (PMC4884025; doi:10.1186/s13568-014-0079-y)
Supplement: Supplementary file 1 — Additional file 1: Normalization of cells/biomass and PHB kinetics from B. thuringiensis culture. Figure S1. Normalization of the biomass kinetics from reported data. The data correspond to different culture systems, different operating conditions, different culture media, different bioreactors, etc., and biomass concentrations were measured using different techniques. The line represents an average biomass kinetic for B. thuringiensis var. kurstaki HD-73. Figure S2. Normalization of the Poly-β-hidroxybutyrate kinetics from reported data. The data correspond to different culture systems, different operating conditions, different culture media, different bioreactors, etc. and Poly-β-hidroxybutyrate concentrations were determined using different techniques. The line represents an average PHB kinetic for B. thuringiensis var. kurstaki HD-73. Figure S3. Effect of the glucose concentration on the Xmax for B. thuringiensis culture (bibliographic data). Figure S4. Effect of the glucose concentration on the tcx for B. thuringiensis culture (bibliographic data). Figure S5. Effect of the glucose concentration on the PHBmax and for B. thuringiensis culture (bibliographic data). Figure S6. Effect of the glucose concentration on the tcp for B. thuringiensis culture (bibliographic data). (PDF 9 MB) [file 13568_2014_79_MOESM1_ESM.pdf]

Supplementary material

AMB Express

Construction of a biodynamic model for Cry protein production studies.

A. Karin Navarro-Mtz[1*] and Fermín Pérez-Guevara[2]

[1] Instituto de Biotecnología, Universidad del Papaloapan, Tuxtepec, Oaxaca, México.
[2] Departamento de Biotecnología, Centro de Investigación y de Estudios Avanzados, México City, México.

* A. Karin Navarro-Mtz, Circuito Central 200, Parque Industrial, 68301 Tuxtepec, Oaxaca, México. Tel. +52 2878759240 ext.220. anavarro@unpa.edu.mx

Normalization of cells/biomass and PHB kinetics from *B. thuringiensis* culture.

The reports to improve the *B. thuringiensis* production process in literature studied several variables and several techniques have been used to measure those variables. Therefore different conditions like culture systems, operating conditions, culture media, bioreactors, etc., were found to influence the productivity and the kinetic parameters of the culture. Concerning the analytic techniques, the dispersion of the reported data can be associated with the methods and techniques used. For example, the most common techniques used for biomass measurement were dry weight, colony-forming unit (CFU), direct count with Neubauer chamber and UV-Vis densitometry. The direct count is the only technique that distinguishes between cells and endospores. Concerning to the Cry protein, several methods to quantify Cry1A(c), including enzyme-linked immunosorbent assay (ELISA), sodium dodecyl sulfate-polyacrylamide gel electrophoresis–densitometry (SDS-PAGE/densitometry), and the total protein assays such as the Bradford or Lowry assay after insecticidal crystal proteins solubilization in alkaline condition have been used.

It is not straightforward to compare the *B. thuringiensis* kinetics data of cells/biomass and PHB reported in the literature because different conditions and analytic techniques used. In order to reduce data to an absolute value that allows us to compare the kinetics reported in the literature, normalization was done. All thirty nine kinetics of cells/biomass and fourteen kinetics of PHB reported in previous studies were normalized. With the normalized data, the analysis of the main variables affecting the three phases of *B. thuringiensis* culture was done.

The normalizations of the cells/biomass kinetics (X_r) were done considering the maximum concentration of cells/biomass (X_{max}) and the time (t_{rx}) when the end of transition phase was observed (t_X) in each experimental data set published, with the Equations (1) and (2).

$$X_r = \frac{X}{X_{max}} \qquad (1)$$

$$t_{rx} = \frac{t}{t_X} \qquad (2)$$

Similarly the normalization of PHB (PHB_r) kinetics were done considering the maximum PHB concentration (PHB_{max}) and the time (t_{rp}) when the deceleration in the PHB production was observed (t_B) with the Equations (3) and (4).

$$PHB_r = \frac{PHB}{PHB_{max}} \qquad (3)$$

$$t_{rp} = \frac{t}{t_B} \qquad (4)$$

The normalization of cells/biomass kinetics reported in literature is show in Figure S1. This figure shows the typical growth kinetic phases for *B. thuringiensis* cultivation: lag phase from 0 to 0.2 of t_{rx}, vegetative growth from 0.2 to 0.7, transition from 0.7 to 1 and sporulation all after. Therefore, the interpretation of the behavior observed in Figure S1 is that cells are growing and multiplying before 1 of t_{rx} and dying thereafter. Biomass measurement techniques like dry weight or UV-Vis densitometry does not distinguish between cells and endospores, thus some growth kinetics does not decrease after 1 of t_{rx}.

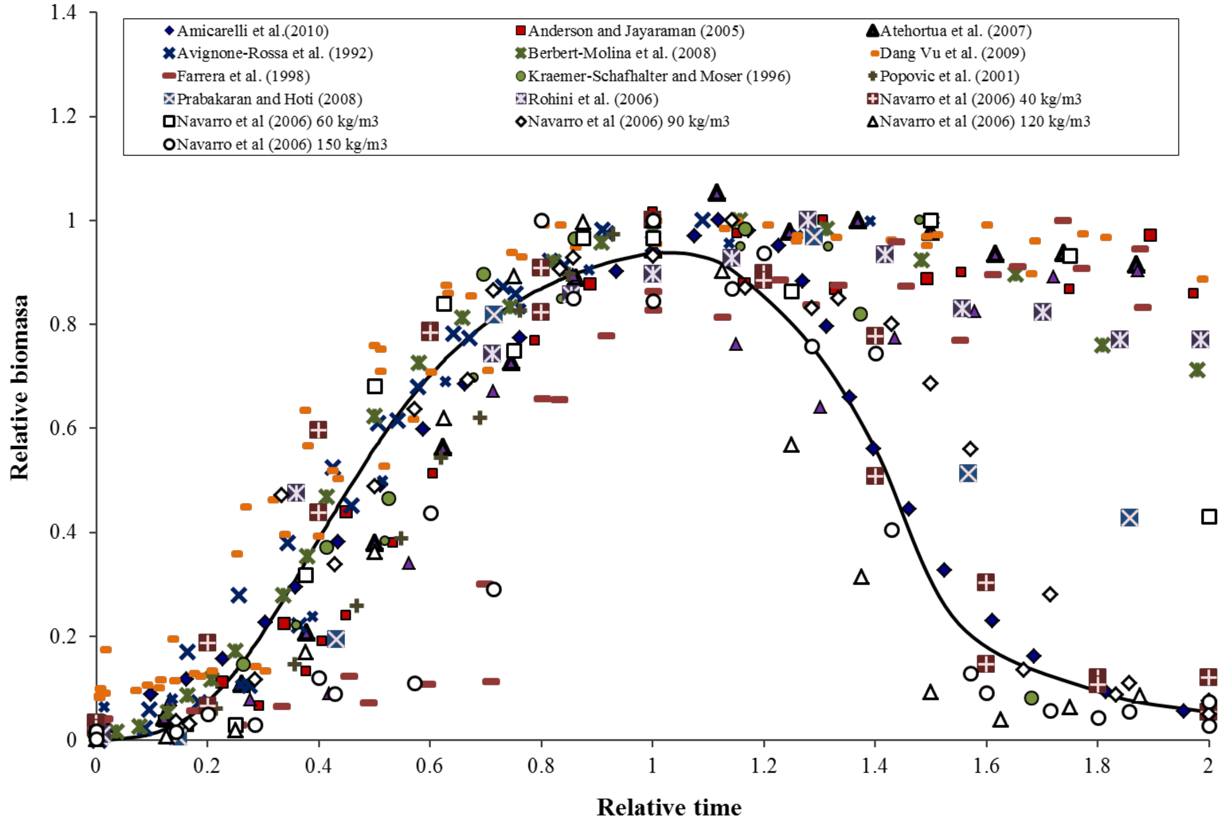

Figure S1. Normalization of the biomass kinetics from reported data. The data correspond to different culture systems, different operating conditions, different culture media, different bioreactors, etc., and biomass concentrations were measured using different techniques. The line represents an average biomass kinetic for *B. thuringiensis* var. *kurstaki* HD-73.

There are few reports in the literature regarding *Bacillus spp.'s* kinetics of PHB production. Figure S2 shows PHB normalization kinetics and the typical PHB kinetic for *Bacillus spp.* cultivation. From 0 to 0.3 of t_{rp}, the PHB_r does not change significantly; but PHB_r increases from 0.3 to 0.8; from 0.8 to 1 the accumulation rate slows down; at t_{rp} the maximum PHB_r concentration is attained followed by a continuous reduction. Therefore, the interpretation of this behavior is that cells are producing PHB before 1 of t_{rp} and consuming it after (Fig. S2). In the case of the PHB consumption kinetics, the behavior is more diverse because some experimental data sets show higher consumption rate than the others, depending on the specific experimental conditions.

The normalization results show that the kinetics of cells/biomass and PHB always have the same behavior and are independent to the culture systems, the operating conditions, the culture media, the bioreactors used or the techniques used to measure.

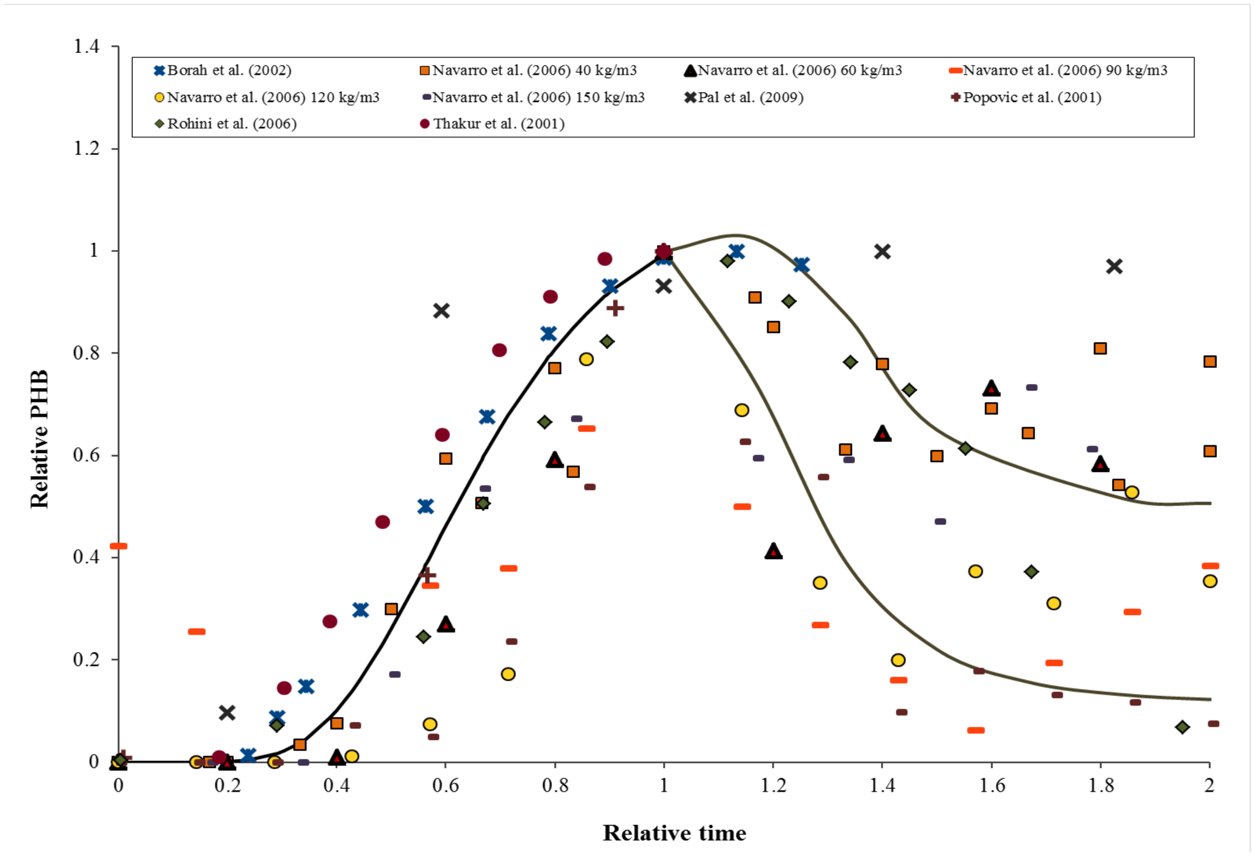

Figure S2. Normalization of the Poly-β-hidroxybutyrate kinetics from reported data. The data correspond to different culture systems, different operating conditions, different culture media, different bioreactors, etc. and Poly-β-hidroxybutyrate concentrations were determined using different techniques. The line represents an average PHB kinetic for *B. thuringiensis* var. *kurstaki* HD-73.

The cells/biomass normalization considers the end of the transition phase as the time when the maximum biomass concentration is reached, this means, when the vegetative growth ends. According to this, in the 20% of the culture time the bacillus is in lag phase, in the 60% is in vegetative growth and in the 20% is in transition phase. For example, if the end of transition phase is reached at the 10 h after inoculation, approximately the first 2 h corresponds to the lag phase, the next 6 h the vegetative growth and the next 2 h the transition phase, after that, the bacilli start to die (from kinetic reported by Navarro et al. 2006). Or if the end of transition phase is reached at the 7 h after inoculation, approximately the first 1.4 h corresponds to the lag phase, the next 4.2 h the vegetative growth and the next 1.4 h the transition phase (from kinetic reported by Popovic et al. 2001).

In the same way, the PHB normalization considers the end of the production phase as the time when the maximum PHB concentration is reached. Therefore, in the 25% of the culture time there is no PHB production, in the 75% the PHB is produced and accumulated, after that, the PHB is consumed. For the same examples that cells/biomass kinetics, if the maximum PHB accumulated is reached at the 10 h after inoculation, approximately the first 2.5 h there is no PHB production and the next 7.5 h the PHB is produced and accumulated (from kinetic reported by Navarro et al. 2006). Or if the maximum PHB accumulated is reached at the 6 h after inoculation, approximately the first 1.5 h there is no PHB production and the next 4.5 h the PHB is produced and accumulated (from kinetic reported by Popovic et al. 2001).

Cell/biomass and PHB kinetics model.

There is not a clear relationship between the culture systems or operation condition or the culture media with the concentration of cells/biomass and PHB. For the analysis to the cells/biomass and PHB kinetics reported in the literature, it

was clear that the principal independent variable is time. Therefore, the maximum concentration of cells/biomass and PHB and the time of the culture are different for all the kinetics. But according to normalization results, cells/biomass and PHB from *B. thuringiensis* cultures have the same behavior, independently of experimental conditions. Also, it is clear that all kinetics data sets present the three phases of the culture; vegetative growth, transition and sporulation and they have the same relation on dimensional time. The normalized kinetics of cells/biomass and PHB shows the typical sigmoidal curve typically well represented by the Gompertz model.

The equation for cells/biomass and PHB was done just for the growth/production phase because most of the kinetics do not present the cell death or PHB consumption data. Although for the model validation, it was considered the growth/production and the death/consumption phase.

Gompertz model for production of vegetative growth phase key compound: cells.

Zwietering et al. (1990) used the Gompertz model to simulate successfully the growth kinetics from several microorganisms. The Gompertz model describes the number of organism as a function of time for growth curves with a lag period and exponential growth (Equation 5). According to these authors, this model contains three parameters (X_{max}, μ_{max} and tc), with a biological meaning, that are sufficient to describe the data. X_{max} (maximal growth value reached) is the maximum concentration of microorganism obtained in the culture; μ_{max} (maximum specific growth rate) is defined as the tangent in the inflection point and is given by the slope of the line when the organisms grow exponentially; and tc (critical time) is the inflection point of the curve and it is defined as the x-axis intercept of this tangent. In general, three parameter models are simpler, easier to use, and more stable than those of four or more parameter due to the less correlation (Zwietering et al. 1990).

$$X = X_{max} e^{\left(-e^{\left(-\mu max(t-tc_x)\right)}\right)} \tag{5}$$

The Gompertz model was used to simulate and estimate the kinetic parameters of the thirty nine kinetics of cells/biomass previously reported. The Gompertz model used for describe growth (Equation 5) was solved with ModelMaker 3.0.3 (Cherwell Scientific, 1997). The model parameters were estimated by nonlinear minimum sum of squares analysis (i.e. the differences between the predicted and measured values) using the Marquadt algorithm.

Gompertz model for production of transition phase key compound: PHB.

The kinetics data sets of PHB obtained from literature correspond to different culture systems, operating conditions, culture media, bioreactors, etc. But the normalization results of PHB production indicates that the PHB kinetics from *Bacillus spp.* have the same behavior, independently to the experimental conditions. The Gompertz model was used to simulate and estimate the kinetic parameters of the fourteen data sets of PHB production from previous studies (Equation 6). Twelve of the reported kinetics were from *B. thuringiensis* and the other two from *B. mycoides* strain. For PHB production kinetics the Gompertz model describes PHB concentration as a function of time. The parameters also have a biological meaning: PHB_{max} (maximal PHB value accumulated) is the maximum PHB concentration accumulated in the culture; μ_{maxp} (maximum specific PHB production rate) and tc_p (critical time for PHB accumulation) are defined in the same way that in the Gompertz model for growth.

$$PHB = PHB_{max} e^{\left(-e^{\left(-\mu_p(t-tc_p)\right)}\right)} \tag{6}$$

The PHB-Gompertz model (Equation 6) was solved with ModelMaker 3.0.3 (Cherwell Scientific, 1997). The model parameters were estimated by nonlinear minimum sum of squares analysis (i.e. the differences between the predicted and measured values) using the Marquadt algorithm.

Gompertz model for sporulation phase key compounds: DPA-Cry protein.

For sporulation key compounds, DPA or Cry no previously reported data could be found. Apparently just a two set of endospores' kinetics formation had been published (Farrera et al. 1998; Navarro et al. 2008). In general the technique used to measure endospores' production is the direct count in Neubauer chamber. This technique has the disadvantage that is really easy to confuse the endospores if solids in suspension are present and then, the measurement error is too high. Navarro et al. (2008) proposed the DPA detection as a more sensitive technique to follow *B. thuringiensis* endospore; this technique considered that DPA is a unique constituent of all endospores. However, the kinetic of *B. thuringiensis* endospore production show a sigmoidal curve according to Farrera et al. (1998) and Navarro et al. (2008). Because Cry protein and the endospores are produce both during the sporulation phase (Bechtel and Bulla, 1976) the beginning of Cry and DPA production occur after the transition phase of the culture, and the main production will be during the dead and lysis of the bacilli so both, Cry and DPA, would be accumulated until the end of the culture. This means that Cry and DPA production will have a lag phase, an exponential production and maximum accumulation. Therefore the Gompertz equation was used to simulate and to estimate the kinetic parameters of both DPA and Cry protein kinetics.

The Gompertz model was statistically sufficient to describe the growth and PHB data for all kinetics. The coefficient of determination for all biomass kinetics data was always greater than 97% ($R^2 = 0.980 \pm 0.054$). The parameters of Gompertz model (X_{max}, μ_{max} and tc) for all kinetics previously reported were evaluated. The microbial growth parameter as biomass concentration (X_{max}) and the maximum specific growth rate (μ_{max}) are essential in many areas of microbiology for example, to characterize effects of antimicrobials, optimize microbial media and to develop kinetic models for use in fermentation technology (Dalgaard and Koutsoumanis 2001). Also those parameters are important for bioreactor design and control. The maximum specific growth rate is the maximal growth capacity and is used to compare growth under different environmental conditions (Molin 1983).

Analysis of cell/biomass and PHB kinetics parameters obtained with the Gompertz model.

The X_{max} and μ_{max} obtained with the Gompertz model for the kinetics previously reported were from 0.69 to 42.71 g/L and from 0.12 to 1.48 1/h, respectively, values that agree with the previously reported (Table I). Although the data reported in previous works were obtained at very different conditions, the X_{max} and tc calculated seem to be related with the initial carbon source concentration in media formulation (Fig. S3 and S4). In contrast, the μ_{max} calculated do not show any dependence with the initial carbon source concentration (data not show). No relationship between the μ_{max} and the X_{max} calculated (data not show) was found. For these results is clear that it is not necessary high growth capacity to generated high concentration of biomass. Furthermore, high carbon sources concentration in the media formulation do not seem to be required to obtain high growth capacities. The formulation and concentration of the substrates in the media and the operating conditions like temperature, pH and dissolved oxygen, change the environment of the bacilli. This environment can affect the physiological state of the bacilli, their metabolic fluxes, rate growth and the time of culture. The μ_{max} calculated shows that the physiological state of the bacilli is different in each culture. With the data analyzed, it was not possible to identify which variables affect μ_{max}.

The critical time (tc) is the inflection point of the curve and indirectly represents the lag phase of the culture. The lag phase is an adjustment period during which bacterial cells modify themselves in order to take advantage of the new environment and initiate exponential growth (Buchanan and Klawitter 1991). As expected, the adaptation time (indirectly represented by tc) for fermentation with high glucose concentration in formulation is greater than those from fermentation with low concentration (Fig. S4).

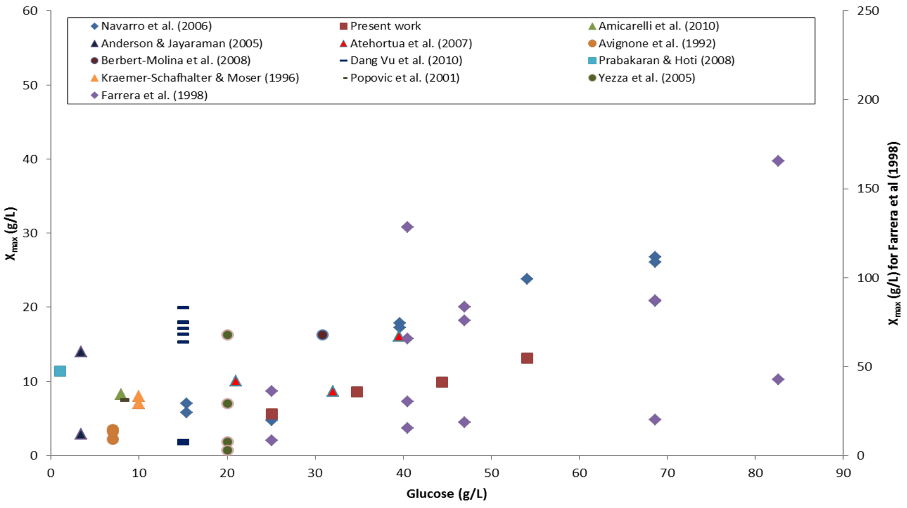

Figure S3. Effect of the glucose concentration on the X_{max} for *B. thuringiensis* culture (bibliographic data).

Similar conditions in the fermentation suggest similar environmental growth conditions and bacilli with similar physiological state. The previously reported fermentation data of Amicarelli et al. (2010), Atehortua et al. (2007), Avignone-Rossa et al. (1992), Farrera et al. (1998), Navarro et al. (2006), Popovic et al. (2001) and Prabakaran and Hotti (2008) have similar production conditions to our fermentation data (batch system with glucose as a principal carbon source at similar operating conditions). For these reports, in Table I and Figs. S3-S4 a clear linear relationship between X_{max} and tc with the glucose concentration in media formulation is observed. However for those data sets no relationship between the μ_{max} and the glucose concentration in media formulation or between X_{max} and μ_{max} could be found. The carbon source most used in *B. thuringiensis* fermentation is glucose and its concentration in the media formulation affects the biomass, endospore and Cry production (Atehortúa et al. 2007; Farrera et al. 1999; Navarro et al. 2006).

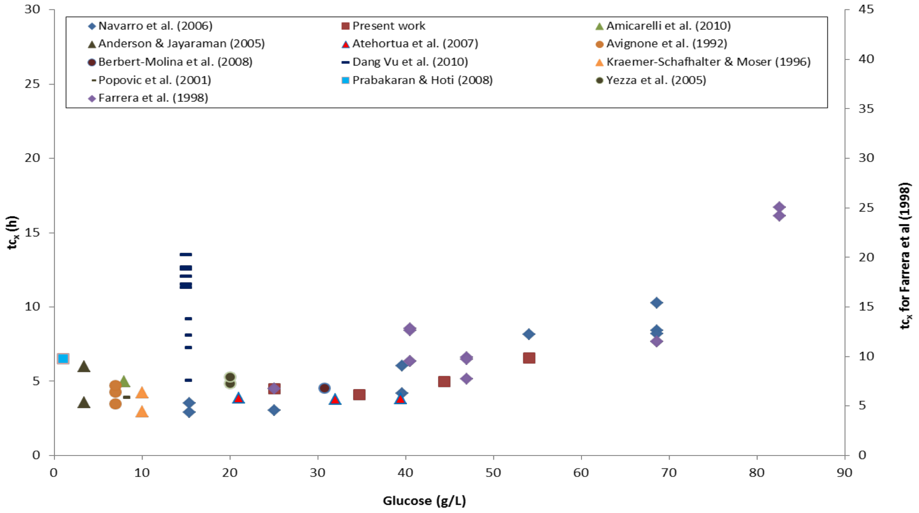

Figure S4. Effect of the glucose concentration on the tc_x for *B. thuringiensis* culture (bibliographic data).

The coefficient of determination for all sets of PHB kinetics data available was always greater than 96% ($R^2 = 0.973 \pm 0.1$). The parameters of PHB production model (PHB_{max}, μ_{PHB} and tc_p) for all kinetics data sets previously reported were evaluated.

The PHB_{max} and μ_{PHB} obtained with the PHB Gompertz model for the kinetics previously reported vary from 0.52 to 3.09 g/L and from 0.078 to 0.92 1/h, respectively. With PHB_{max} data is not possible to evaluate the PHB percentage of cell dry weight because the authors do not normally reported the cells/biomass kinetic. Similarly as for growth model, the PHB_{max} and tc_p calculated apparently are related with the carbon source concentration in media formulation (Fig. S5 and S6). In contrast, the μ_{PHB} calculated do not show any dependence with the carbon source concentration in media formulation (data not show). No relationship between the μ_{PHB} and the PHB_{max} calculated was found (data not show). In literature just one report was found with growth and PHB kinetics data for the same experiments (Popovic et al. 2001).

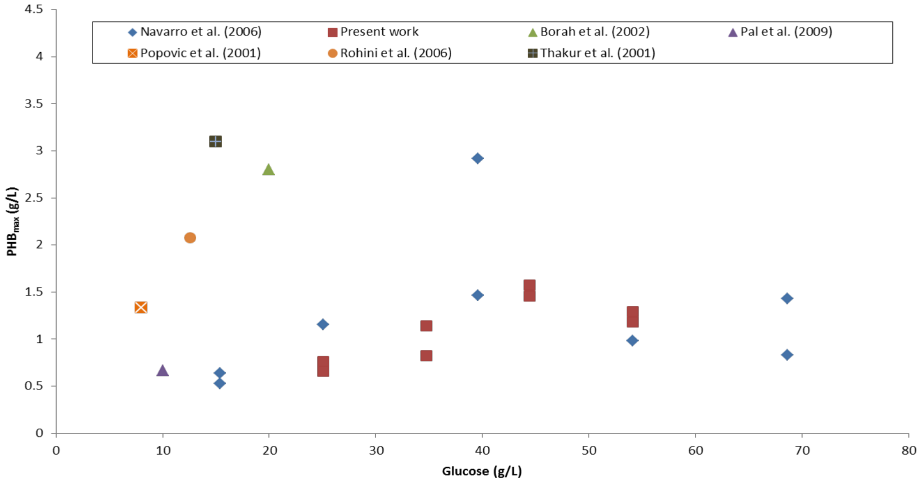

Figure S5. Effect of the glucose concentration on the PHB_{max} and for *B. thuringiensis* culture (bibliographic data).

The PHB is produced during vegetative growth and transition phase then, as expected, tc_p is greater than tc. The difference between tc and tc_p, also was observed in normalization figures (Fig. S1 and S2). The tc_p for fermentations with high glucose concentration in formulation is greater than for fermentations with low substrate concentration (Fig. S6).

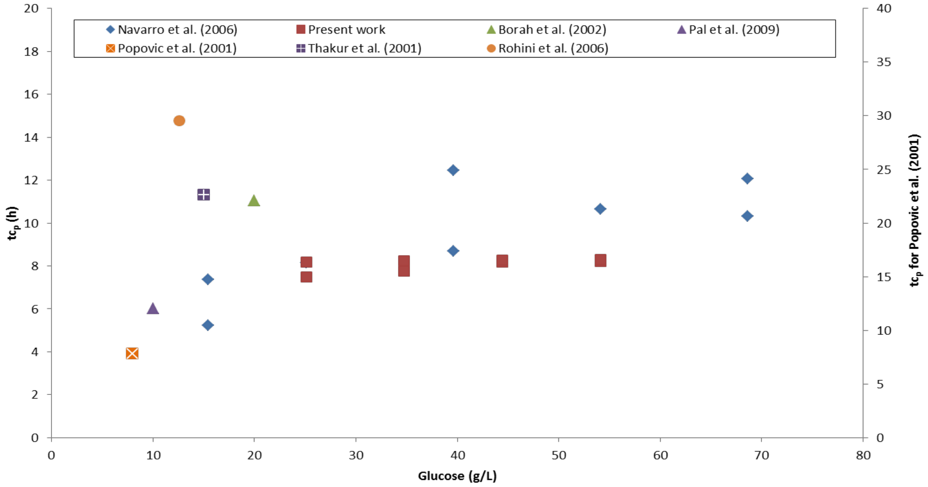

Figure S6. Effect of the glucose concentration on the tc_p for *B. thuringiensis* culture (bibliographic data).

References.

Amicarelli A, di Sciascio F, Toibero JM, Alvarez H (2010) Including dissolved oxygen dynamics into the *Bt* δ-endotoxins production process model and its application to process control. Braz J Chem Eng 27:41-62

Atehortúa P, Álvarez H, Orduz S (2007) Modeling of growth and sporulation of *Bacillus thuringiensis* in an intermittent fed batch culture with total cell retention. Bioprocess Biosyst Eng 30:447–456

Avignone-Rossa C, Arcas J, Mignone C (1992) *Bacillus thuringiensis* growth, sporulation and δ-endotoxin production in oxygen limited and non-limited cultures. World J Microbiol Biotechnol 8:301-304

Bechtel DB, Bulla LA (1976) Electron microscope study of sporulation and parasporal crystal formation in *Bacillus thuringiensis*. J Bacteriol 127:1472-1481

Borah B, Thakur PS, Nigam JN (2002) The influence of nutritional and environmental conditions on the accumulation of poly-β-hydroxybutyrate in *Bacillus mycoides* RLJ B-017. J Appl Microbiol 92:776-783

Buchanan RL, Solberg M (1972) Interaction of sodium nitrite, oxygen and pH on growth of *Staphylococcus aureus*. J Food Sci 37:81– 85

Dalgaard P, Koutsoumanis K (2001) Comparison of maximum specific growth rates and lag times estimated from absorbance and viable count data by different mathematical models. J Microbiol Methods 43:183–196

Dang Vu K, Tyagi RD, Valero JR, Surampalli RY (2010) Batch and fed-batch fermentation of *Bacillus thuringiensis* using starch industry wastewater as fermentation substrate. Bioproc Biosyst Eng 33:691–700

Farrera RR, Pérez-Guevara F, de la Torre M (1998). Carbon:nitrogen ratio interacts with initial concentration of total solids on insecticidal crystal protein and spore production in *Bacillus thuringiensis* HD-73. Appl Microbiol Biotechnol 49:758-765

Molin G (1983). Measurement of the maximum specific growth rate in chemostat of *Pseudomonas spp.* with different abilities for biofilm formation. Eur J Appl Microbiol Biotechnol 18:303-307

Navarro AK, Farrera RR, Lopez R, Perez-Guevara F (2006) Relationship between poly-β-hydroxybutyrate production and δ-endotoxin for *Bacillus thuringiensis* var. *kurstaki*. Biotechnol Lett 28:641-644

Navarro AK, Peña A, Pérez-Guevara F (2008) Endospore-Dipicolinic acid detection during *Bacillus thuringiensis* culture. Lett Appl Microbiol 46:166-170

Popovic M, Liu W, Iannotti EL, Bajpai RK (2001) A mathematical model for vegetative growth of *Bacillus thuriniensis*. Eng Life Sci 2:85-90

Prabakaran G, Hoti SL (2008) Influence of amino nitrogen in the culture medium enhances the production of δ-endotoxin and biomass of *Bacillus thuringiensis* var. *israelensis* for the large-scale production of the mosquito control agent. J Ind Microbiol Biotechnol 35:961–965

Thakur PS, Borah B, Baruah SD, Nigam JN (2001) Growth-associated production of Poly-3-hydroxybutyrate by *Bacillus mycoides*. Folia Microbiol 46:488-494

Yezza A, Tyagi1 RD, Valéro JR, Surampalli, RY (2005) Wastewater sludge pre-treatment for enhancing entomotoxicity produced by *Bacillus thuringiensis* var. *kurstaki*. World J Microbiol Biotechnol 21: 1165–1174

Zwietering MH, Jongenburger I, Rombouts FM, Van'T Riet K (1990) Modeling of bacterial growth curve. Appl Envirom Microbiol 56:1875-1881
