# Supplementary figures and images for: Construction of a biodynamic model for Cry protein production studies
Source: AMB Express. 2014 Nov 14;4:79. doi: 10.1186/s13568-014-0079-y (PMC4884025; doi:10.1186/s13568-014-0079-y)

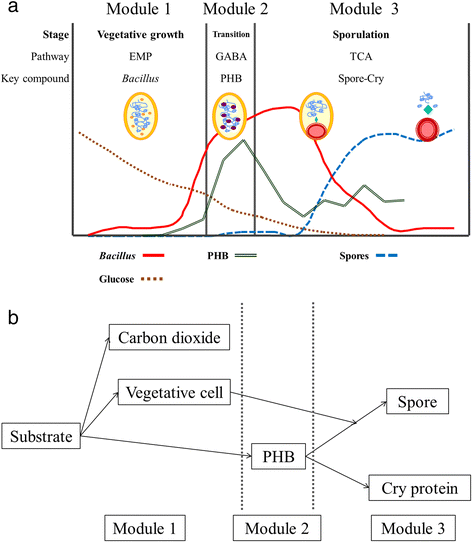

Supplement: Supplementary file 2 — Authors’ original file for figure 1 [file 13568_2014_79_MOESM2_ESM.gif]

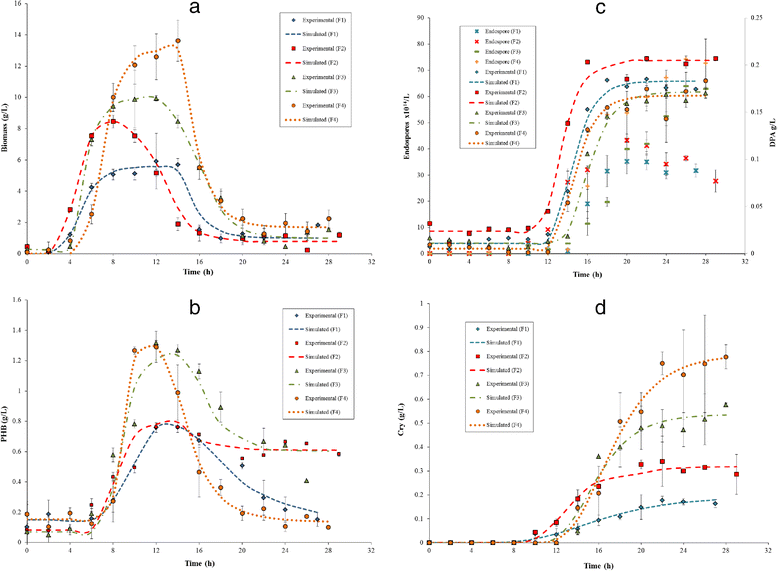

Supplement: Supplementary file 3 — Authors’ original file for figure 2 [file 13568_2014_79_MOESM3_ESM.gif]

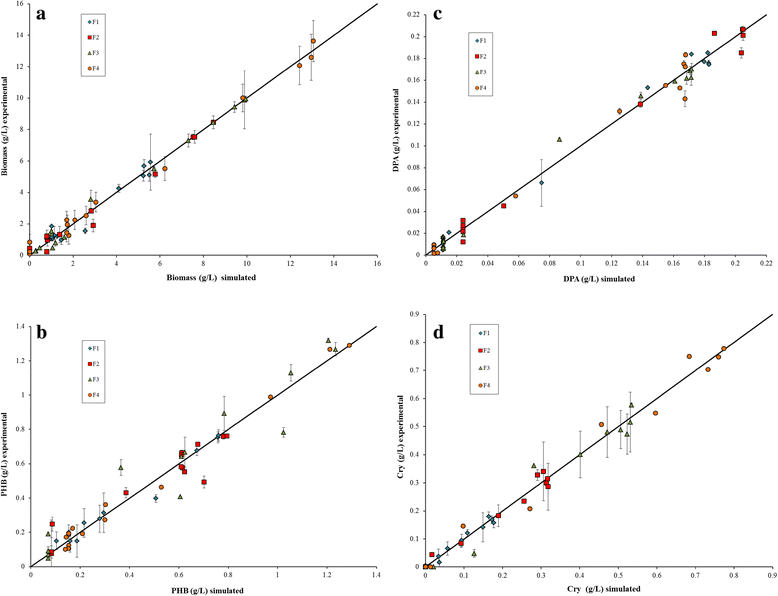

Supplement: Supplementary file 4 — Authors’ original file for figure 3 [file 13568_2014_79_MOESM4_ESM.gif]

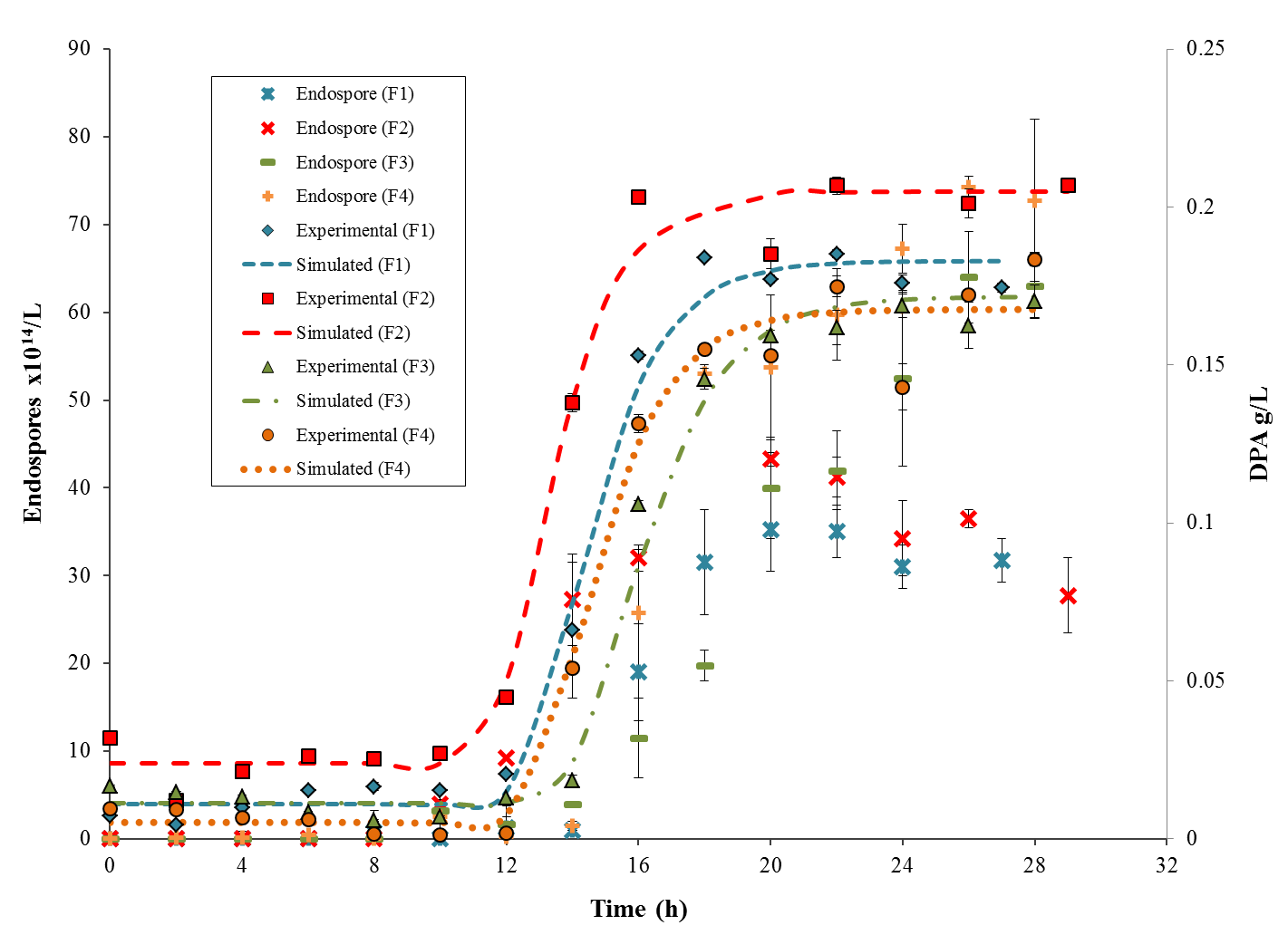

Supplement: Supplementary file 5 — Authors’ original file for figure 4 [file 13568_2014_79_MOESM5_ESM.tiff]

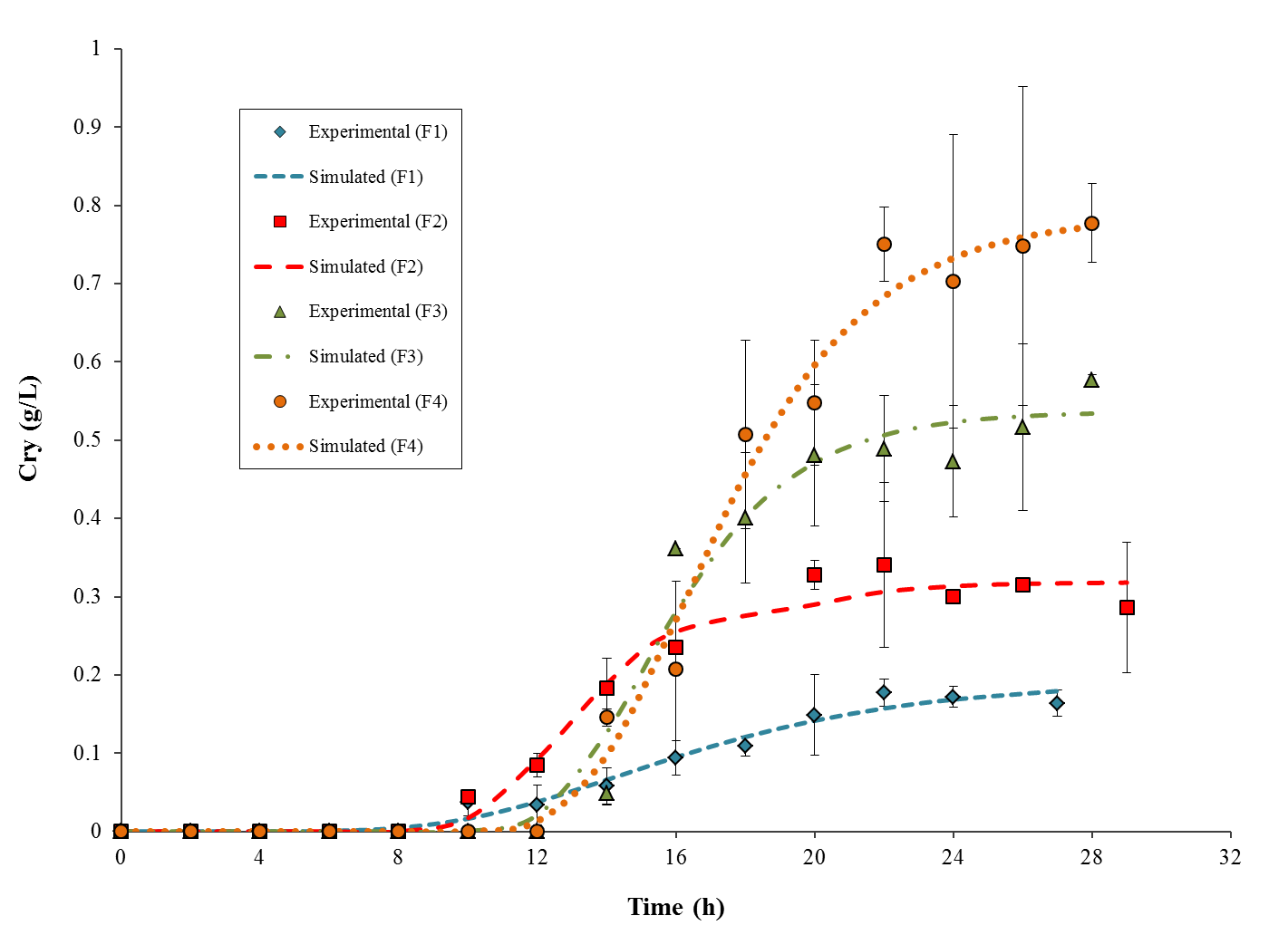

Supplement: Supplementary file 6 — Authors’ original file for figure 5 [file 13568_2014_79_MOESM6_ESM.tiff]

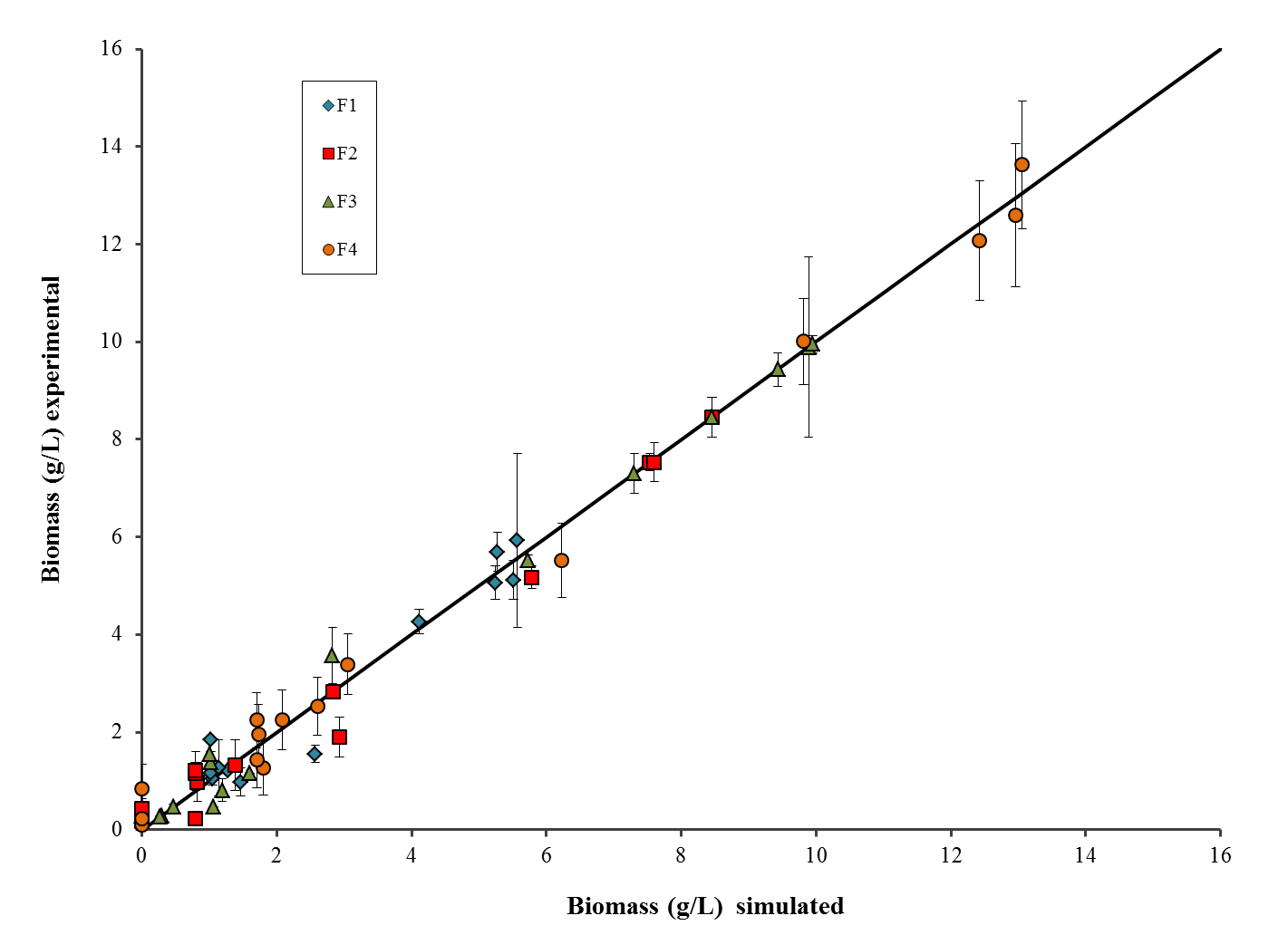

Supplement: Supplementary file 7 — Authors’ original file for figure 6 [file 13568_2014_79_MOESM7_ESM.tiff]

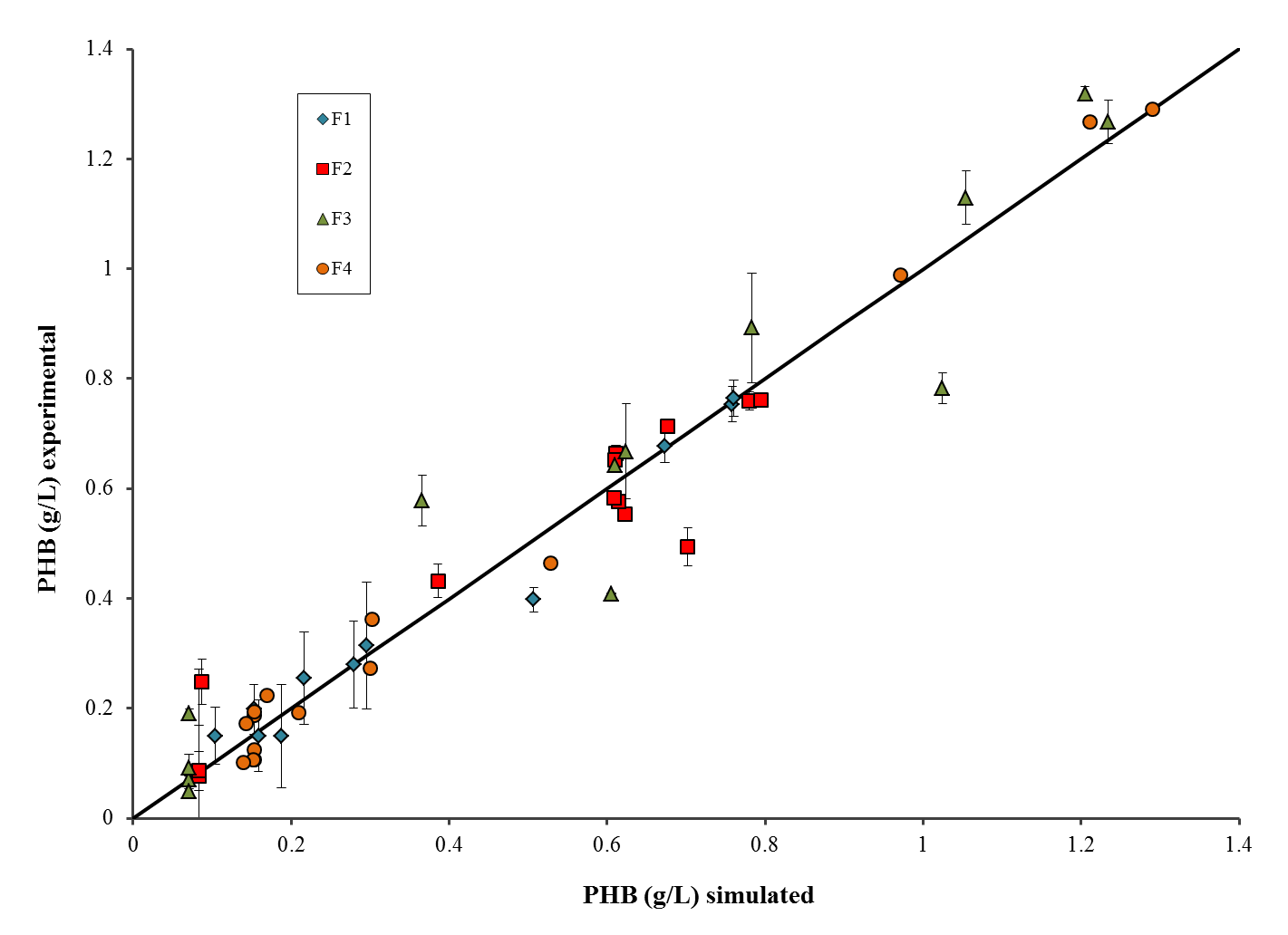

Supplement: Supplementary file 8 — Authors’ original file for figure 7 [file 13568_2014_79_MOESM8_ESM.tiff]

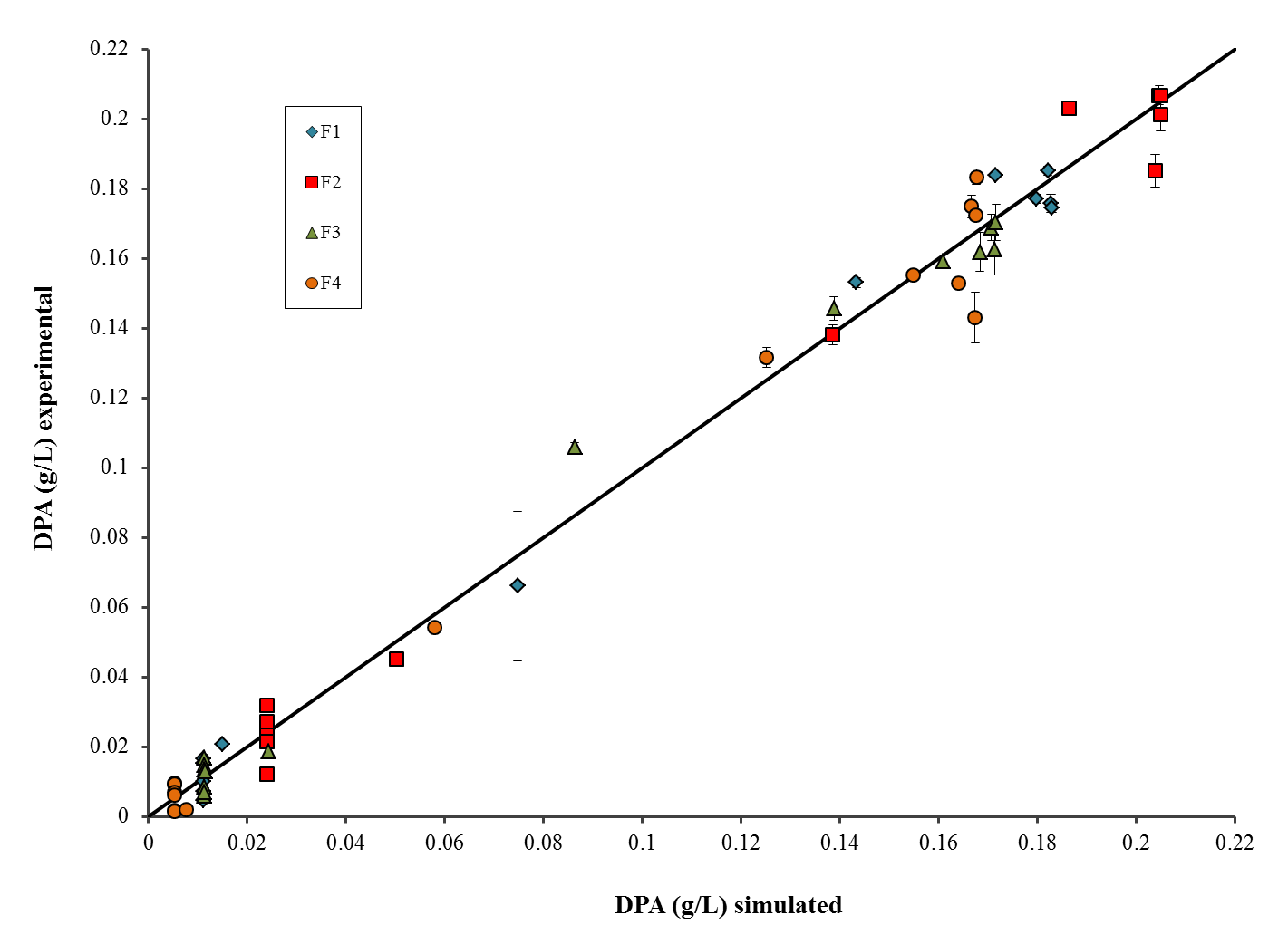

Supplement: Supplementary file 9 — Authors’ original file for figure 8 [file 13568_2014_79_MOESM9_ESM.tiff]

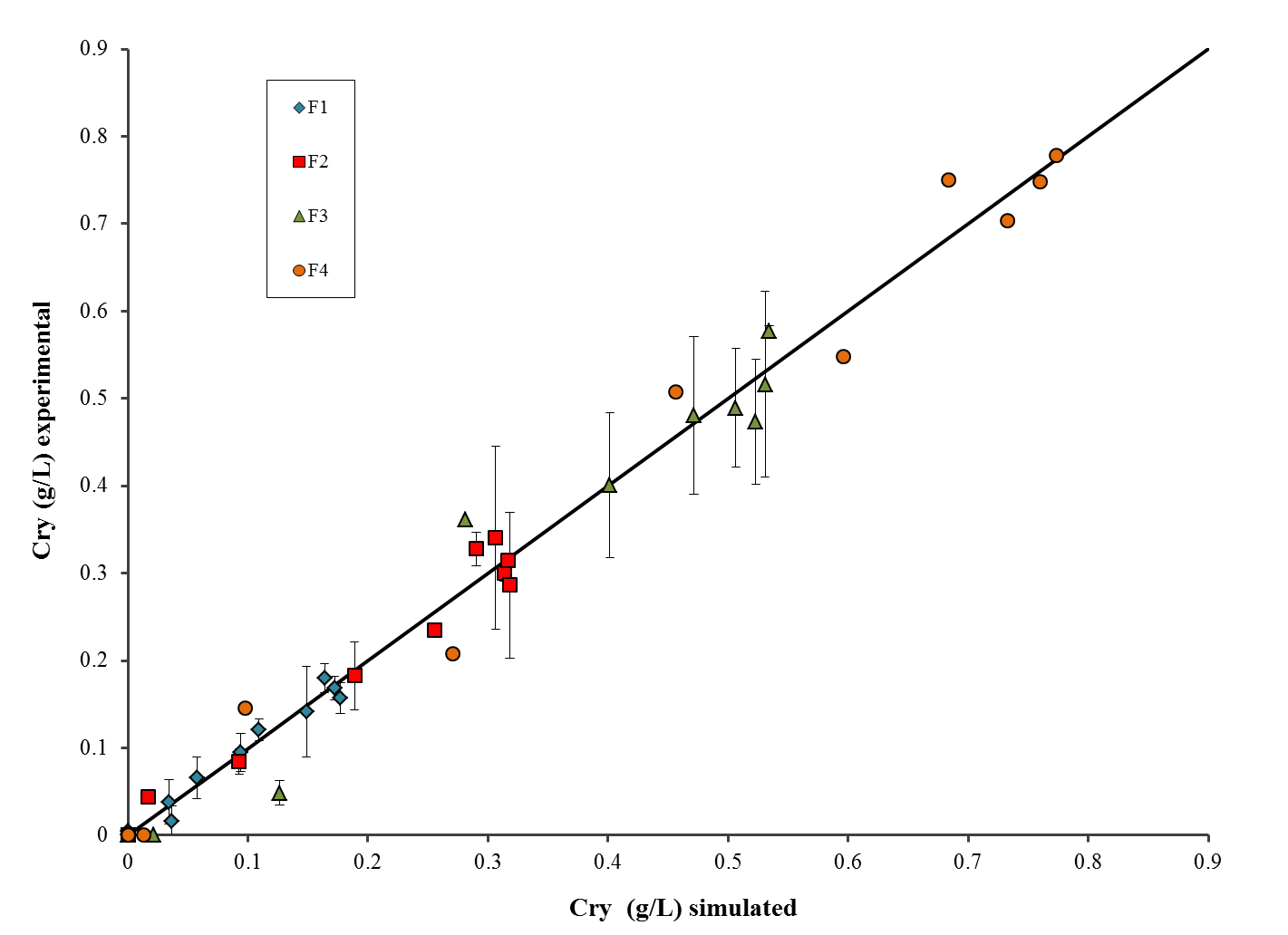

Supplement: Supplementary file 10 — Authors’ original file for figure 9 [file 13568_2014_79_MOESM10_ESM.tiff]
